# Transcription factor scleraxis vitally contributes to progenitor lineage direction in wound healing of adult tendon in mice

**DOI:** 10.1074/jbc.RA118.001987

**Published:** 2018-03-05

**Authors:** Tomoya Sakabe, Keiko Sakai, Toru Maeda, Ataru Sunaga, Nao Furuta, Ronen Schweitzer, Takako Sasaki, Takao Sakai

**Affiliations:** From the ‡ Medical Research Council Centre for Drug Safety Science, Department of Molecular and Clinical Pharmacology, Institute of Translational Medicine, University of Liverpool, Liverpool L69 3GE, United Kingdom,; § Department of Biomedical Engineering, Lerner Research Institute, Cleveland Clinic, Cleveland, Ohio 44195,; ¶ Research Division, Shriners Hospital for Children, Portland, Oregon 97239, and; ‖ Department of Biochemistry, Faculty of Medicine, Oita University, Oita 879-5593, Japan

**Keywords:** extracellular matrix, tendon, wound healing, mouse genetics, cell biology, transforming growth factor β (TGF-β), scleraxis, tissue progenitor cells, conditional knockout

## Abstract

Tendon is a dense connective tissue that transmits high mechanical forces from skeletal muscle to bone. The transcription factor scleraxis (Scx) is a highly specific marker of both precursor and mature tendon cells (tenocytes). Mice lacking *scx* exhibit a specific and virtually complete loss of tendons during development. However, the functional contribution of Scx to wound healing in adult tendon has not yet been fully characterized. Here, using *ScxGFP*-tracking and loss-of-function systems, we show in an adult mouse model of Achilles tendon injury that paratenon cells, representing a stem cell antigen-1 (Sca-1)–positive and Scx-negative progenitor subpopulation, display Scx induction, migrate to the wound site, and produce extracellular matrix (ECM) to bridge the defect, whereas resident tenocytes exhibit a delayed response. Scx induction in the progenitors is initiated by transforming growth factor β (TGF-β) signaling. *scx*-deficient mice had migration of Sca-1–positive progenitor cell to the lesion site but impaired ECM assembly to bridge the defect. Mechanistically, *scx*-null progenitors displayed higher chondrogenic potential with up-regulation of SRY-box 9 (Sox9) coactivator PPAR-γ coactivator-1α (PGC-1α) *in vitro*, and knock-in analysis revealed that forced expression of full-length *scx* significantly inhibited *Sox9* expression. Accordingly, *scx*-null wounds formed cartilage-like tissues that developed ectopic ossification. Our findings indicate a critical role of Scx in a progenitor-cell lineage in wound healing of adult mouse tendon. These progenitor cells could represent targets in strategies to facilitate tendon repair. We propose that this lineage-regulatory mechanism in tissue progenitors could apply to a broader set of tissues or biological systems in the body.

## Introduction

Tendon is a dense connective tissue rich in extracellular matrix (ECM),^2^ and it transmits high contraction forces from skeletal muscle to bone due to dense, robust, and highly organized parallel and longitudinal collagen bundles (1–3). Resident tendon cells (“tenocytes”), like skeletal myoblasts, chondrocytes, and osteoblasts, originate during embryogenesis from multipotent mesenchymal cells and actively produce unique and tendon-specific ECM (4). Adult tendon injury, overuse, and age-related degeneration are a difficult clinical problem that occurs frequently. Injured tendon heals very slowly and is rarely restored to its normal undamaged state in terms of its structural integrity. The reasons behind the slow-healing response to injury and the exact nature of the cells responsible for the complex repair process are still poorly understood (5–7).

Scleraxis (Scx), a basic helix-loop-helix (HLH) transcription factor, was recently identified as a highly specific marker of tenogenic precursor cells and mature differentiated tenocytes during development (8, 9). Mice lacking the *scx* gene are viable but show a specific and virtually complete loss of major force-transmitting and intermuscular tendons (10). Scx is thus the master gene shown to be crucial for the tendon lineage. The examination of changes in Scx expression offers an ideal tool for analyzing alterations in adult tendon tissue remodeling.

We have previously demonstrated that the expression of Scx in tenocytes is regulated by transforming growth factor β (TGF-β)/Smad2/3–mediated signaling (11). Tenocytes in tendons/ligaments with osteoarthritis acquire chondrogenic potential; *e.g.* they show down-regulation of Scx and up-regulation of the chondrocyte marker SRY-box 9 (Sox9), strongly suggesting that chondrogenic differentiation is associated with the progression of degeneration in tendons/ligaments (12). Although these two transcription factors, Scx and Sox9, coordinately regulate the determination of cellular lineages during embryonic development (13), their precise contribution to lineage specifications and the signaling pathways in lineage specifications are both still largely unknown. Furthermore, no comprehensive study to explore the functional role of Scx following adult tendon injury has yet been carried out. Tendon stem/progenitor cells have been shown to exist in adult normal human and mouse tendon (14), and they localize in both tendon proper (within the endotenon) and peritenon (including paratenon and epitenon) (15, 16). It remains to be elucidated what cell type plays the main role in adult tendon healing/remodeling following injury and how Scx regulates these tendon-cell phenotypes during these processes. Nevertheless, no studies to date identify a definitive requirement for Scx in response to adult tendon injury.

In the present study, we used a combination of *ScxGFP*-tracking and loss-of-function systems to explore whether Scx is a suitable molecular target for accelerating the healing response to adult tendon injury. We have taken advantage of *ScxGFP* transgenic mice, which express the marker green fluorescent protein (GFP) driven by *scx* regulatory sequences such that this allows one to track tenocytes at any time following adult tendon injury using ScxGFP as a marker (9, 11). Because complete deletion of the *scx* gene during development results in severe tendon-defect phenotypes (10), we have utilized adenovirus-*Cre* to induce deletion of the *scx* gene only in injured adult tendon tissues. Here, we show a critical role of Scx in adult tendon progenitor cell lineage in the repair following tendon injury.

## Results

### Cells in the paratenon are involved in repair following adult Achilles tendon injury

**Figure 1.**
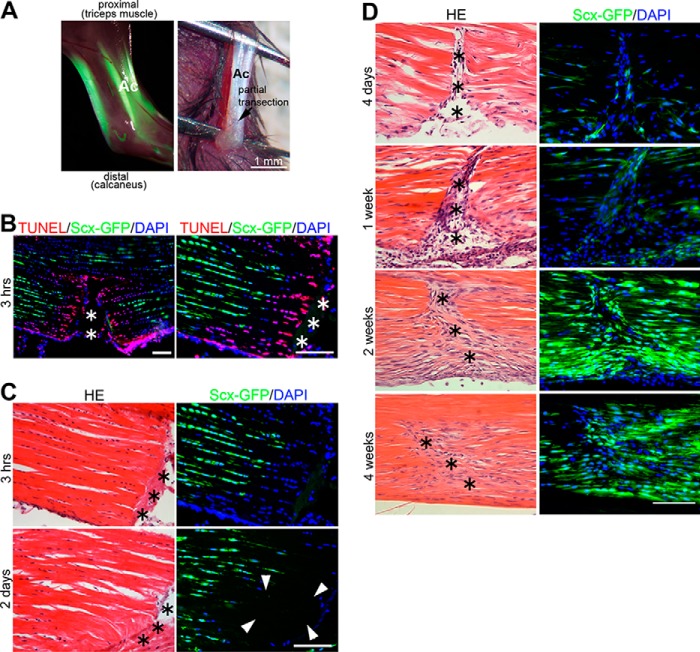
**Adult tendon wound healing after the partial transection in *ScxGFP* mouse Achilles tendons.** *A*, partial transection of Achilles tendon. *Left panel*, a normal Achilles tendon (*Ac*) in 10-week-old *ScxGFP* transgenic mice expresses a robust ScxGFP signal (*green*) under fluorescence stereomicroscopy. *Right panel*, partial transection of adult mouse Achilles tendon (*Ac*) during the operation. *Scale bar*, 1 mm. *B*, analysis of cell death at 3 h after partial transection at lower (*left panel*) and higher (*right panel*) magnification. Triple staining for TUNEL (*red*), ScxGFP (*green*), and DAPI (cell nuclei; *blue*) is shown. *Asterisks* indicate wounds. *Scale bar*, 100 μm. *C* and *D*, histological analysis of Achilles tendon in the partial transection model at 3 h and 2 days after injury (in the initial inflammatory stage) (*C*) and 4 days and 1, 2, and 4 weeks after injury (in the proliferation stage) (*D*). H&E (*HE*) staining is shown in the *left panels*, and the same area with a GFP/UV filter is shown in the *right panels* (*green*, ScxGFP; *blue*, nuclear staining with DAPI). *Asterisks* in H&E sections indicate wounds. An acellular region is formed surrounding the wound at day 2 (*C*; *arrowheads*). From 1 to 2 weeks after operation, a continuous cellular stream is formed from the peritendinous region to the wounded site, and these cells start expressing ScxGFP. *Scale bar*, 100 μm.

The sudden loss of tensile loading in the complete Achilles tendon transection model induces an excessive release of active TGF-β and causes massive tendon cell death (11). This model is not suitable for assessment of the contribution, if any, of resident tenocytes to adult tendon wound healing. The complete tendon transection model is also known to result in chondroid degeneration/ossification at the edges in addition to regeneration in the center of injured tendons following injury (17–20). Therefore, we developed a simple and reproducible Achilles tendon “partial transection” model in which tensile loading from skeletal muscles is not completely lost (Fig. 1*A*). To track the healing process following tendon injury in a dynamic fashion, we studied the tissue distribution of tenocytes and the expression levels of Scx using ScxGFP as a tenocyte marker. Indeed, a majority of resident tenocytes (∼94% of total cells were ScxGFP-positive) in adult Achilles tendons from *ScxGFP* transgenic mice robustly expressed ScxGFP (Fig. S1) (11).

Tendon wound healing involves both regeneration of tenocytes and reconstruction of ECMs, which are mainly composed of collagen fibrils. ECM repair is known to proceed through three overlapping stages: an initial inflammatory stage (typically spanning a few days postinjury), a proliferative stage (starting a few days post injury), and a remodeling stage (starting ∼1–2 months postinjury) (for reviews, see Refs. 5 and 21). Because tensile loading is important for the maintenance of Scx expression in adult tenocytes (11), we hypothesized that partial transection of the Achilles tendon affects its expression of Scx. In the inflammatory phase following partial transection, a marked decrease in the number of ScxGFP-positive cells was already observed at 3 h postinjury, and terminal deoxynucleotidyltransferase dUTP nick end labeling (TUNEL)–positive cells were detected (Fig. 1, *B* and *C*). This region had become virtually acellular by day 2 (∼16.0% of 4′,6-diamidino-2-phenylinodole (DAPI)–positive cells were ScxGFP-positive; Fig. 1*C*, *arrowheads*). In the proliferative phase, marked cellular hyperplasia of the paratenon close to the wounded region was observed. Those cells showed a continuous cellular stream from paratenon to wounded site at 1 week following injury (Fig. 1*D*, *HE* section), and ∼46.7% of those cells (84 of 180 DAPI-positive cells) were found to express ScxGFP (Fig. 1*D*, *ScxGFP/DAPI* section). At 2 weeks following injury, the majority of those cells (156 of a total of 184 DAPI-positive cells; ∼84.8%) manifested as a series of robustly ScxGFP-expressing cells in the wound region (Fig. 1*D*), and there was a continuous, intense ScxGFP-positive cellular stream from paratenon to wounded site (hereafter called “wound ScxGFP cells”) (Fig. S2). The wound was filled up with those cells by 4 weeks (Fig. 1*D*).

### ECM remodeling in adult tendon wound healing is different from that in other tissues

**Figure 2.**
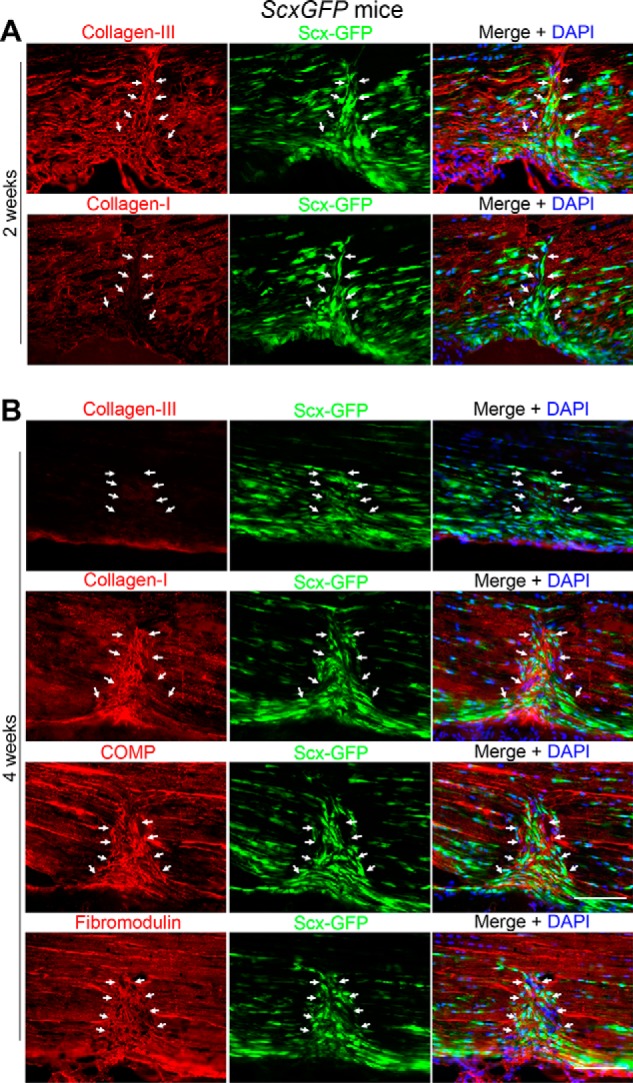
**Deposition of ECMs in wound site at 2 (*A*) and 4 weeks (*B*) after operation.** Shown are collagen type III, collagen type I, COMP, and fibromodulin (*left panels*; *red*), ScxGFP (*middle panels*; *green*), and merged images plus DAPI (*blue*) (*right panels*). Collagen type III is produced and bridges the wound sites at 2 weeks, and type III fibrils are replaced by type I fibrils by 4 weeks. *Arrows* indicate the wounded area. *Scale bar*, 100 μm.

ECM remodeling during tendon wound healing is thought to follow the same processes as in other tissues. In general, a provisional matrix is initially formed with the plasma proteins fibrinogen and fibronectin, which triggers fibroblast migration. Fibroblasts that have migrated to the wound site play a major role in remodeling the ECM followed by replacement of the provisional matrix by collagen type III and then type I fibrils (22, 23). Following injury by partial transection of the Achilles tendon, however, there was little exudation of fibrinogen and little angiogenesis (as judged by the endothelial-cell marker platelet endothelial cell adhesion molecule-1 (PECAM-1 (CD31))) in the wounded region by 1 week due to the largely avascular nature of adult tendon (Ref. 5 and Fig. S3). In ECM remodeling following injury, collagen type III is synthesized as procollagen III in a similar manner to collagen type I. However, whereas most of the N-propeptide of the procollagen type I molecule is cleaved, the type III N-propeptide is processed partially (24). We tracked the assembly and cellular source of collagen type III using anti-type III N-propeptide antibody. Immunofluorescence analysis revealed that the deposition of collagen type III was evident in the ECM of the wound site, and it formed a collagenous bridge over the defect space at 2 weeks following injury (Fig. 2*A*, *arrows*). Those collagen type III fibrils were overlapped with wound ScxGFP cells in the wounded site (∼61.7% of total DAPI-positive cells), suggesting wound ScxGFP cells as a cellular source in collagen production (Fig. 2*A*). The collagen type III fibrils deposited had been gradually replaced by *de novo* collagen type I fibrils by 4 weeks, whereas at 2 weeks very little deposition of collagen type I was found at the wound site (Fig. 2, *A* and *B*). The deposition of the small leucine-rich proteoglycan fibromodulin and cartilage oligomeric matrix protein (COMP; or thrombospondin 5), which play regulatory roles in collagen matrix assembly (25, 26), was also observed at 4 weeks after injury (Fig. 2*B*). The deposited ECMs and cells in the wound site often showed a pattern aligned perpendicular to the tendon axis (Fig. 2). Because *Scx* is not expressed in nontendon adult fibroblasts (11), these findings suggest that, unlike other tissues, wound ScxGFP cells could be the principal source of cells that reconstruct the damaged ECM following tendon injury.

### Tendon progenitor cells that reside in the paratenon differentiate into ScxGFP-expressing tenocytes and contribute as a main player to tendon wound healing

**Figure 3.**
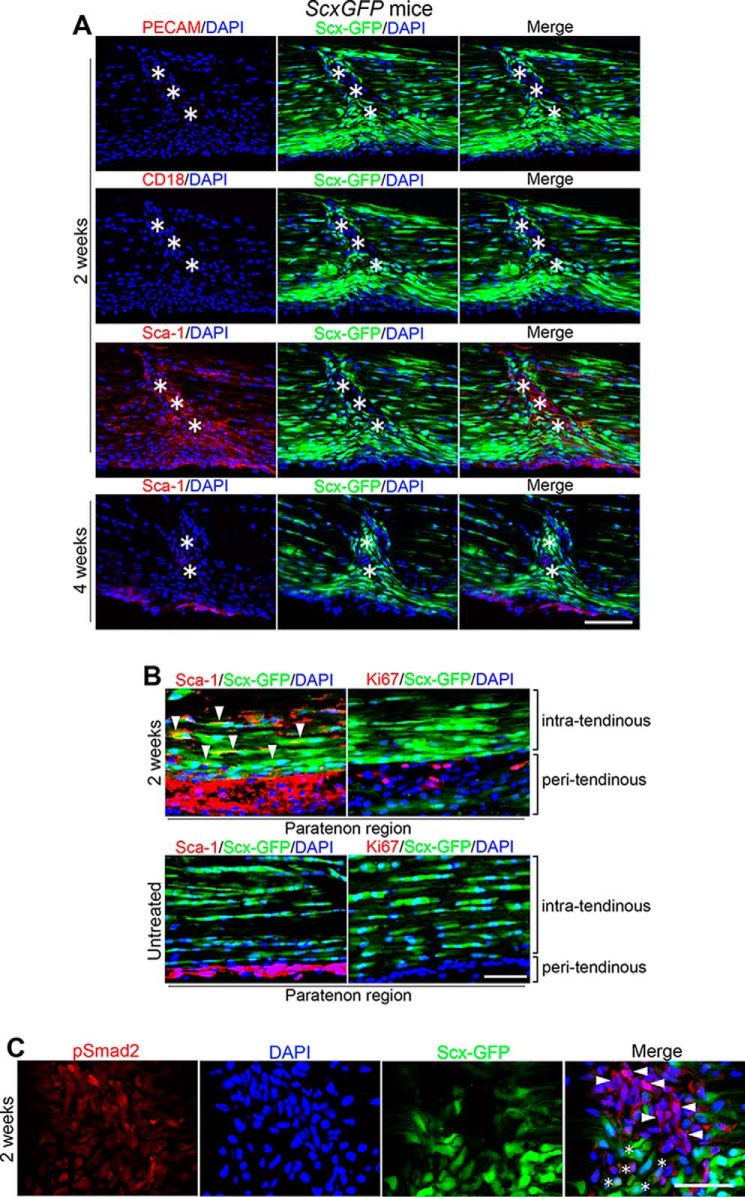
**Characterization of cells migrating to the wound site.** *A*, PECAM/DAPI, CD18/DAPI, or Sca-1/DAPI (*left panels*; PECAM, CD18, and Sca-1, *red*; DAPI, *blue*); ScxGFP/DAPI (*middle panels*; ScxGFP, *green*; DAPI, *blue*); and merged images (*right panels*). The migrating cells express tendon progenitor cell marker Sca-1 but are negative for endothelial cell marker PECAM and BMSC marker CD18 at 2 weeks after injury. These Sca-1–positive cells show induced ScxGFP expression and down-regulated Sca-1 expression by 4 weeks after injury. *Asterisks* indicate the wounded area. *Scale bar*, 100 μm. *B*, tissue distribution of Sca-1–positive cells in the paratenon region of adult wound (at 2 weeks after injury; *upper panels*) and untreated (*lower panels*) Achilles tendon. Triple immunofluorescence staining for Sca-1 (*red*)/ScxGFP (*green*)/DAPI (*blue*) (*left panels*) and Ki67 (*red*)/ScxGFP (*green*)/DAPI (*blue*) (*right panels*) is shown. Sca-1–positive cells localize in the peritendinous region in the untreated tendons. Following injury, a notable expansion of Sca-1–positive cells is observed with some Ki67-positive cells, and these cells migrate into the intratendinous region with the induction of Scx as evidenced by Sca-1/ScxGFP double–positive cells (*arrowheads*). *Scale bar*, 50 μm. *C*, triple immunofluorescence staining for (from *left* to *right*) pSmad-2 (*red*), DAPI (*blue*), ScxGFP (*green*), and merged image in the wounded site of Achilles tendon at 2 weeks after injury. Cells show nuclear expression of pSmad2 (*arrowheads*), and some are pSmad2/ScxGFP double–positive (*asterisks*). *Scale bar*, 50 μm.

Next, we determined the cellular origin of wound ScxGFP cells, which are significant players following tendon injury. In the adult complete Achilles tendon transection model, extrinsic cells have been suggested to play a role in scar formation following injury (20). Such cells may be derived from (i) vascular endothelial cells (27); (ii) bone marrow–derived stromal cells (BMSCs), which have been shown to migrate into damaged tissues in response to injury or inflammation (28); or (iii) *in situ* tendon progenitor cells (14). Immunostaining for the endothelial cell marker PECAM showed no significant cellular distribution at 2 weeks following injury (Fig. 3*A*). Immunostaining for CD18, a surface receptor integrin β2 chain present on BMSCs, showed very scattered positive cell distribution in the wounded tendon at 2 weeks (Fig. 3*A*). Bone marrow transplantation analysis further confirmed that the cells that had migrated from bone marrow to the tendon wound site comprised less than 15% of the total cells (14.9 ± 3.9% at 1 week and 11.2 ± 3.2% at 2 weeks following injury; Fig. S4), suggesting that BMSCs did not have a major contribution to adult tendon wound healing.

Tendon progenitor cells have been shown to exist in adult mouse tendons. They express stem cell antigen-1 (Sca-1) and CD44 but are negative for CD18, a surface receptor integrin β2 chain present on BMSCs (14). In a study of the tissue distribution of progenitor cells in adult untreated Achilles tendon, the progenitor cells were found to localize in the paratenon region, showing a characteristic pattern of cell-surface markers: Sca-1–positive as a progenitor marker, Ki67-negative as a proliferation marker, and ScxGFP-negative (Fig. 3*B*). In contrast, a notable expansion of Sca-1–positive cells was observed in the markedly thickened paratenon region at 2 weeks following injury as evidenced by marked numbers of Sca-1–positive cells and some Ki67-positive cells, and we identified Sca-1/ScxGFP double–positive cells in the intratendinous region (Fig. 3*B*). We hypothesized that those progenitors contributed as major players in tendon wound healing. At 2 weeks following injury, there was a continuous Sca-1–positive cellular stream from paratenon to wound site (Fig. 3*A*), and importantly, ∼22.4% of total DAPI-positive cells were Sca-1/ScxGFP double–positive in the wound site (Fig. 3*A*). There were very few Ki67-positive cells in the intratendinous region (data not shown).

Resident mature tenocytes existing originally in tendon tissues (tendon proper) may contribute to repair following injury. Indeed, we found those cells as a minor population within adjacent tendon struts following injury (Fig. S2). Those cells were aligned parallel to the tendon axis with rounded/polygonal morphology. They expressed tyrosine 397–phosphorylated focal adhesion kinase and activated integrin β1, suggesting active migration in response to injury (Fig. S5). However, those cells showed a delayed response compared with wound ScxGFP cells: unlike wound ScxGFP cells at 1 and 2 weeks (Fig. 1*D*, *HE* at 1 week and *ScxGFP* at 2 weeks), those resident tenocytes did not have a continuous cellular stream to the wounded site and were distributed away from the wound with lower expression levels of ScxGFP. Taking these findings together, we concluded that (i) Sca-1–positive tendon progenitor cells localized in the paratenon likely migrate to the wound site, differentiate into ScxGFP-expressing tenocytes, function as the principal cell type involved in adult tendon healing, and actively produce collagen fibrils to repair the damaged tendon and (ii) resident Scx-expressing tenocytes are also likely to migrate to the wound site but only in the later stages after the progenitor cells have made their contribution.

### Isolation, generation, and characterization of mouse adult tendon progenitor cell lines

**Figure 4.**
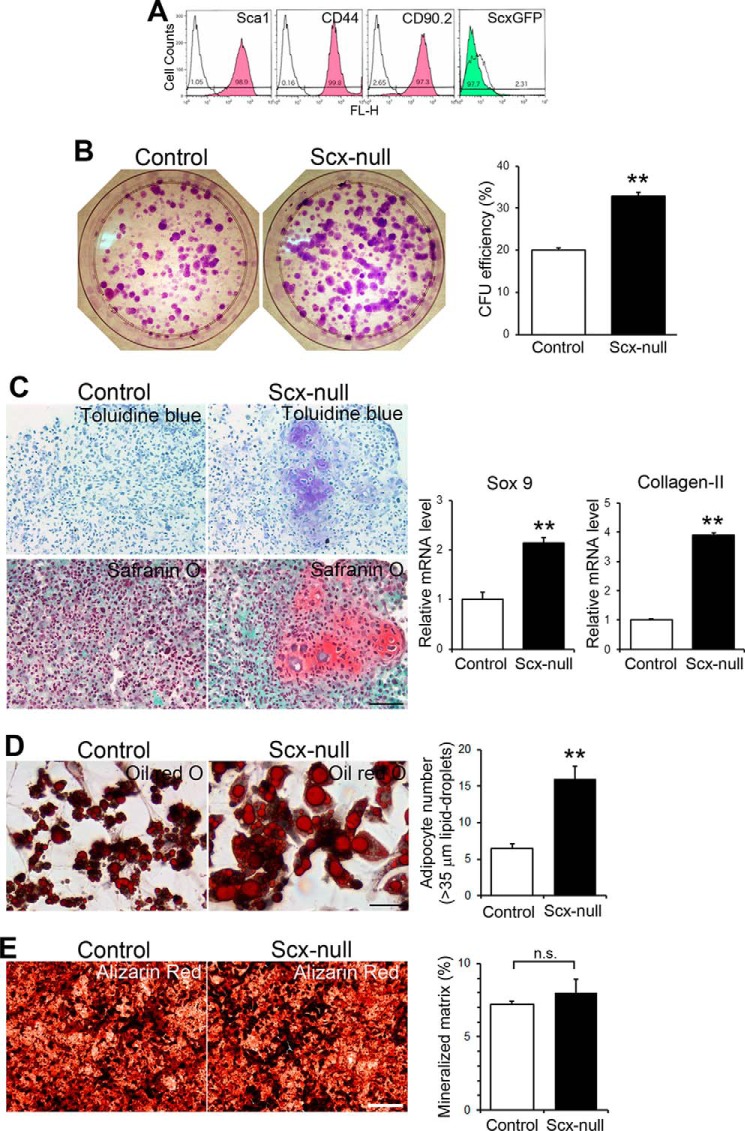
**Isolation, generation, and characterization of mouse adult parental and *scx*-null tendon progenitor cell lines.** *A*, expression profiles of cell-surface markers in sorted *scx^flox^*^/^*^flox^*/*ScxGFP*/*p21*^−/[minus^] cells by FACS analysis. *B–E*, characterization of *scx*-null and its parental adult tendon progenitor cells. *B*, self-renewal analysis. *Left panel*, colonies formed visualized by crystal violet at day 10. *Right panel*, cfu assay in *scx*-null and its parental progenitor cells. *Error bars* represent the standard deviation (*n* = 3 for each group). **, *p* < 0.01. *C*, chondrogenic differentiation. (Left panels) Toluidine blue and Safranin O staining at day 21. *scx*-null cultures show cartilage-like tissues. *Scale bar*, 100 μm. *Right panels*, real-time PCR analysis of *Sox9* and type II collagen mRNA levels at day 7 after differentiation. Relative mRNA expression levels are shown relative to the control value of 1. *Error bars* represent the standard deviation (*n* = 6 for each group). **, *p* < 0.01. *D*, adipogenic differentiation. *Left panel*, Oil Red O staining at day 21. *scx*-null cultures show larger lipid droplet–containing adipocytes. *Scale bar*, 100 μm. *Right panel*, number of adipocytes containing lipid-droplets more than 35 μm in diameter. *Error bars* represent the standard deviation (*n* = 4 for each group). **, *p* < 0.01. *E*, osteogenic differentiation. *Left panel*, Alizarin Red staining at day 21. *Scale bar*, 500 μm. *Right panel*, Areas of formation of mineralized and amorphous substances. *Error bars* represent the standard deviation (*n* = 4 for each group). *n.s.*, not significant.

Next, to investigate further the role of Scx in tendon progenitor cell phenotypes, we generated progenitor cell lines from adult *scx^flox^*^/^*^flox^*/*ScxGFP* mouse Achilles tendon under a *Trp53*- and *Cdkn1a* (*p21*)–null genetic background (23). Flow cytometry analysis to study cell-surface markers showed that the progenitor cell lines generated were positive for the stem cell marker Sca-1 (98.9% positivity), CD44 (99.8%), and CD90.2 (97.3%); had very low levels of the bone marrow stromal cell marker CD18 (∼3%), the endothelial cell marker CD31 (∼3%), the leukocyte marker CD45 (∼3%), and ScxGFP (2.3%); and had low expression for the stem cell marker CD34 (6%) (Fig. 4*A* and data not shown) (14).

TGF-β is the most potent cytokine to induce ScxGFP expression in adult resident tenocytes *in vitro* (11). A recent transcriptomic analysis of mouse embryonic limb tendon cells during development has shown that TGF-β signaling via Smad2/3 is necessary and sufficient to drive mouse mesodermal cell differentiation toward the tendon lineage (29). Ten candidate molecules have been examined for a role in the induction of *Scx* expression in adult tendon progenitors (16, 30–34). TGF-β1, -β2, and -β3 were the most potent and were able to induce ScxGFP expression *in vitro*, although TGF-β2 was less potent than TGF-β1 and -β3 (Fig. S6A). However, the expression level of ScxGFP in progenitors in response to TGF-β1 was markedly lower (∼67.2%; *p* < 0.01) than in adult tenocytes (Fig. S6B). Neither the osteoinductive cytokine BMP2 (35), used as a negative control, nor any other cytokine/growth factor examined had any effect (Fig. S6A). In TGF-β signaling, the ligand TGF-β binds to cell-surface TGF-β receptors. On binding of the ligand, receptor complexes initiate downstream Smad signaling pathways (36, 37). Indeed, some cells in the continuous cellular stream from paratenon to the wound site at 2 weeks following tendon injury were found to express nuclear phospho-Smad2, and some of them were phoshpo-Smad2 (pSmad2)/ScxGFP double–positive (Fig. 3*C*). These findings suggest that cytokine TGF-β could participate in the differentiation of adult tendon progenitors into ScxGFP-expressing tenocytes during adult tendon wound healing.

### Scx-null tendon progenitors display significantly higher chondrogenic potential in vitro

The parental progenitor clones established as described above were subsequently treated with a Cre-transducing adenovirus to delete the *scx^flox^*^/^*^flox^* genes, and *scx*-null progenitor cell lines were generated (Fig. S7). We then explored whether the loss of the *scx* gene leads to alteration of progenitor cell self-renewal potential by cfu assay. Interestingly, the *scx*-null progenitor cells formed significantly more colonies (∼1.56-fold increase) than parental cells at day 10 (*p* < 0.01; Fig. 4*B*), although parental progenitor cells were found to be negative for ScxGFP expression (Fig. 4*A*).

To investigate further whether the differentiation potential of tendon progenitor cells is affected by the absence of Scx, cells were subjected to adipogenic, chondrogenic, and osteogenic differentiation analyses. Indeed, *scx*-null and its parental progenitor cells successfully differentiated into the three lineages (Fig. 4, *C–E*). When progenitor cells were transferred to micromass cultures with TGF-β to induce chondrogenic differentiation (38), *scx*-null progenitor cells showed significantly higher mRNA levels of chondrogenic markers such as *Sox9* and type II collagen from an early stage (day 7) of chondrogenic differentiation (Fig. 4*C*). At day 21, *scx*-null cell cultures showed cartilage-like tissue formations (Fig. 4*C*). Although the tendon progenitor cells from fibromodulin/biglycan double–null mice exhibit down-regulation of *scx* expression (14), *scx*-null progenitors did not show significant alteration of fibromodulin or biglycan mRNA levels compared with parental controls when stimulated to differentiate toward the chondrogenic lineage (data not shown). In adipogenesis, the accumulation of intracellular lipids is used as a marker of adipocyte differentiation (39): *scx*-null cultures developed adipocytes that contained significantly larger lipid droplets as evidenced by Oil Red O staining. The number of adipocytes that contained lipid droplets more than 35 μm in diameter was 16.0 ± 3.6 cells/field in *scx*-null cultures *versus* 6.5 ± 1.3 cells/field in parental cultures (*n* = 4; field = 0.25 mm^2^; *p* < 0.01; Fig. 4*D*). In osteogenesis, the production of mineralized and amorphous substances was increased in *scx*-null cells compared with control parental cells as evidenced by Alizarin Red staining, although the areas of mineralized matrix formed did not show a significant difference statistically (8.00 ± 1.8% in *scx*-null cultures *versus* 7.23 ± 0.4% in parental cultures (*n* = 4; field = 1.6 mm^2^; *p* = 0.45; Fig. 4*E*). Furthermore, the expression levels of pSmad1/5/8 protein and runt-related gene-2 (*runx2*) mRNA in the presence of the osteogenesis inducer BMP2 (14, 40) were similar between *scx*-null and parental cell cultures (Fig. S8). Taken together, these findings indicate that the loss of Scx results in enhanced differentiation potential of tendon progenitor cells.

### The absence of Scx results in a defect in progenitor differentiation into Scx-expressing tenocytes and tendon repair

To further explore the functional role of Scx in adult tendon wound healing, we developed here an Achilles tendon-specific *scx*-conditional knockout model in adult mice. Although we found that ScxGFP-expressing tenocytes originating from tendon progenitor cells played a significant role in the early stages (at least for up to 28 days) following Achilles tendon injury (Fig. 1 and Fig. S2), we could not exclude the possibility that resident tenocytes expressing Scx participate in the healing process at certain time points, *e.g.* a later remodeling stage. To circumvent this, we utilized a *scx^flox^*^/^*^flox^* mouse strain, and an adenovirus encoding *Cre* recombinase was injected locally into wounded tendons to inactivate the *scx* gene. To study the efficiency of adenovirus encoding *Cre* recombinase in the deletion of the target gene, we performed initial studies using mice harboring the well characterized *ROSA26^flox^*^/^*^flox^-LacZ* gene. This gene expresses a bifunctional LacZ/neomycin phosphotransferase protein in any cell (and cell progeny) once recombined by Cre (41). We found that a single injection of adenovirus-*Cre* (1 × 10^9^ multiplicity of infection (m.o.i.) in 10 μl of PBS) into the Achilles tendon of *ROSA26* mice showed efficient gene recombination in tenocytes at 1 week after injection (Fig. S9A). No endogenous LacZ expression was found in the Achilles tendon of WT mice (data not shown). We also gave a single injection of 1 × 10^9^ m.o.i. adenovirus-*Cre* into the Achilles tendon of adult *scx^flox^*^/^*^flox^* mice and confirmed considerably decreased expression of Scx protein in tenocytes by immunohistochemistry at 1 week after injection (Fig. S9B), confirming that this model resulted in the efficient deletion of floxed alleles in the *scx* gene. No decrease in tenocyte numbers was observed when adenovirus-GFP was injected as a control (data not shown). Thus, we established an adult mouse model in which inactivation of the *scx* gene was induced locally and specifically in all cells from Achilles tendons.

**Figure 5.**
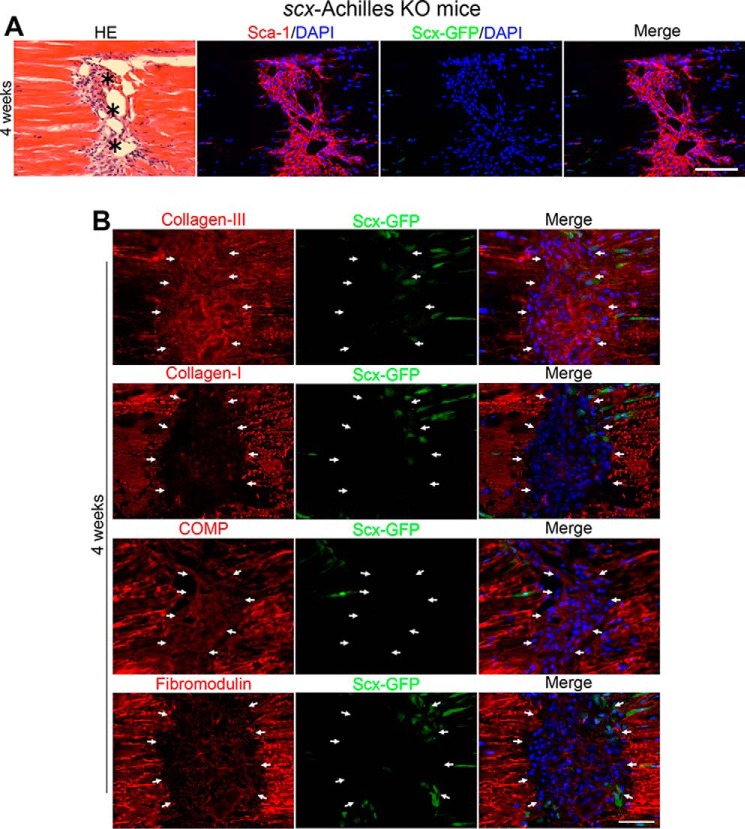
**Tendon wound does not heal without *scx*.** *A*, H&E (*HE*) and immunofluorescence staining for Sca-1 (*red*)/DAPI (*blue*), ScxGFP (*green*)/DAPI (*blue*), and merged image (from *left* to *right*) in *scx*-Achilles KO mice at 4 weeks following injury. *Asterisks* in sections indicate wounds. *Scale bar*, 100 μm. *B*, deposition of ECMs in wounded site of *scx*-Achilles KO mice at 4 weeks after injury. Shown are collagen type III, collagen type I, COMP, and fibromodulin (*left panels*; *red*); ScxGFP (*middle panels*; *green*); and merged images plus DAPI (*blue*) (*right panels*). Collagen type III fibrils are not replaced by collagen type I fibrils at 4 weeks. *Arrows* indicate the wound area. *Scale bar*, 100 μm.

Next, to delete the *scx* gene at the time of tendon injury in adult *scx^flox^*^/^*^flox^*/*ScxGFP* transgene mice, we pretreated Achilles tendons with adenovirus-*Cre* at 1 × 10^9^ m.o.i. for 20 min (*scx*-Achilles KO mice) and then partially transected the Achilles tendons. The wounds in adenovirus-*Cre*–untreated control mice were filled with ScxGFP-expressing tendon cells and built up with *de novo* collagen type I fibrils by day 28 (Fig. 2*B*). In contrast, although the wounds in *scx*-Achilles KO mice showed migration of paratenon-derived Sca-1–positive progenitors to the wound site at 4 weeks after injury, those cells failed to undergo down-regulation of Sca-1 and induction of ScxGFP during migration from paratenon to the wound site and still retained high expression levels of Sca-1 (Fig. 5*A*). Moreover, the cells that had migrated to the wound site showed abnormal deposition patterns of ECMs at 4 weeks after injury (Fig. 5*B*). In the control wound, the deposited collagen type III in the wounded site was gradually replaced with type I collagen fibrils from 2 to 4 weeks following injury (Fig. 2). In contrast, in mutant wounds, the deposited collagen type III was not replaced with collagen type I by 4 weeks (Fig. 5*B*). Interestingly, the mutant wound also lacked deposition of the small leucine-rich proteoglycan fibromodulin and COMP at 4 weeks (Figs. 5*B* and 2*B*).

**Figure 6.**
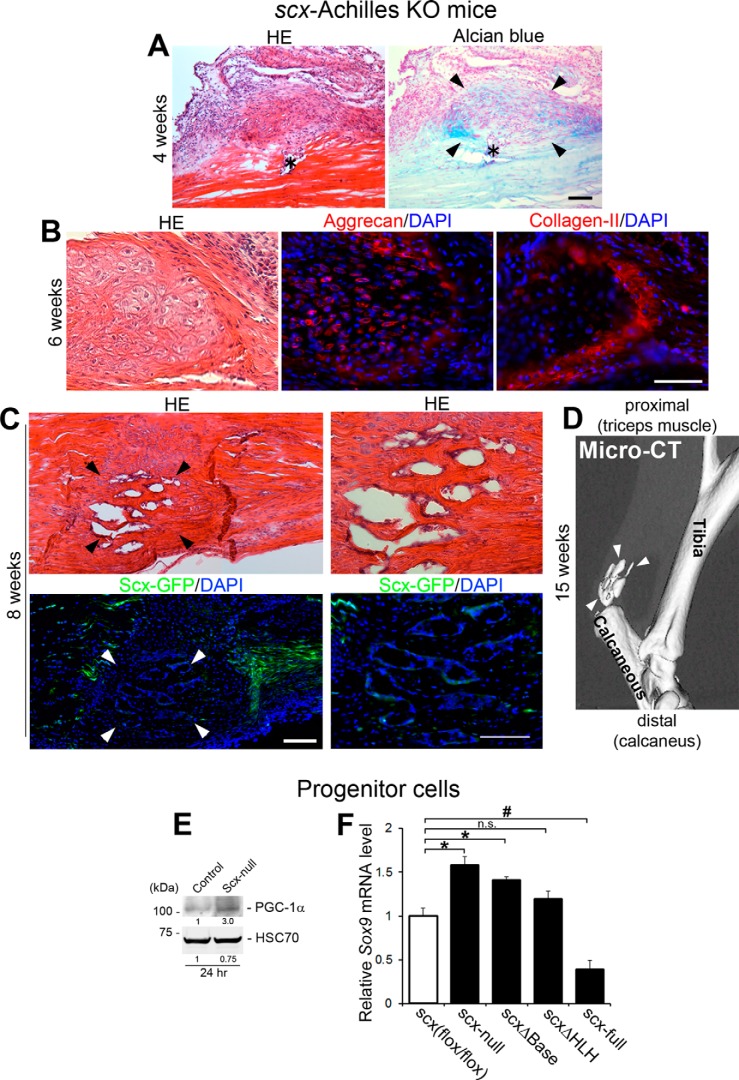
**Ectopic ossification in the long-term follow up of *scx*-Achilles KO mice following tendon injury.** *A–D*, cartilage-like tissue formation and ectopic ossification in mutant Achilles tendon following injury. *A*, ectopic cartilage-like foci (*arrowheads*) are formed around the wound site of mutant wounds at 4 weeks following injury. *Asterisks* indicate wounds. *Scale bar*,100 μm. *B*, histological analysis of Achilles tendon of mutant mice at 6 weeks following injury. Serial sections stained for H&E (*HE*) (*left panel*), aggrecan (*red*)/DAPI (*blue*) (*middle panel*), and collagen type II (*red*)/DAPI (*blue*) (*right panel*). *Scale bar*, 100 μm. *C*, histological analysis of Achilles tendon of mutant mice at 8 weeks following injury. *Left panels*, H&E staining (*left upper panel*) and the same area with GFP/UV filter (l*eft lower panel*; *green*, ScxGFP; *blue*, DAPI). *Right panels*, higher magnification of areas indicated with *arrowheads. Scale bars*, 100 μm. *D*, micro-CT analysis at 15 weeks after injury. *Arrowheads* indicate the foci of calcification. *E* and *F*, *scx*-null progenitor cells show significant chondrogenic potential *in vitro. E*, Western blot analysis of PGC-1α protein expression in *scx*-null and parental (control) progenitor cells at 24 h after treatment with chondrogenic differentiation medium. The band intensities were measured by densitometry, and the intensity of the control sample value was set to 1. Each value is shown relative to this control value. The positions of molecular mass marker are indicated. *F*, real-time PCR analysis of *Sox9* mRNA levels at 5 days after treatment with chondrogenic differentiation medium in *scx^flox^*^/^*^flox^* and *scx*-null tenocytes and tenocytes overexpressing full-length Scx (*scx*-full), DNA-binding domain–deleted Scx (*scx*Δ*Base*), or helix-loop-helix protein interaction domain–deleted Scx (*scx*Δ*HLH*). Relative mRNA expression level is shown relative to the control value of 1. *Error bars* represent the standard deviation (*n* = 3 for each group). *, significantly up-regulated (*p* < 0.05); #, significantly down-regulated (*p* < 0.05); *n.s.*, not significant.

Subsequent histopathological analyses revealed Alcian blue–positive foci composed of chondrocyte-like cells in mutant wounds from 4 weeks following injury (Fig. 6*A*). Such foci were observed in all mutant wounds (six of six), whereas no foci were found in control wounds. Because *scx*-null tendon progenitors displayed significantly higher chondrogenic potential *in vitro* (Fig. 4*C*), we hypothesized that tendon progenitor derivatives lacking Scx had indeed undergone a fate change and possessed chondrogenic potential *in vivo*. As expected, ECMs in the mutant wounded site were composed of the cartilage markers collagen type II and aggrecan (Fig. 6*B*). It is known that ectopic mineralization can be caused by the endochondral ossification program (42). Consistent with these interpretations, in a longer-term followup, such foci demonstrated ectopic ossification from 8 weeks and were surrounded by chondrocyte-like cells (Fig. 6*C*). Microcomputed tomography (micro-CT) analysis clearly showed ectopic ossification foci in the mutant wound at 15 weeks following injury (Fig. 6*D*), indicating that the absence of Scx causes the phenotypes of tendon progenitors to switch to a genuine chondrocyte cell fate *in vivo*. Neither cartilage formation nor ectopic ossification Dfoci were observed in partial transection wounds in control mice. No significant phenotypic differences were observed between mice that had received no adenovirus and those that had received the control adenovirus (data not shown), ruling out any major cytotoxic effect of the viral solution itself. PBS-injected and sham-operated Achilles tendons did not show any disease phenotype (data not shown).

Next, the functional link between Scx and Sox9-mediated chondrogenesis was further addressed *in vitro*. There is evidence that peroxisome proliferator–activated receptor (PPAR) γ coactivator-1α (PGC-1α) acts as a coactivator for Sox9 to regulate cartilage-specific transcriptional activities during chondrogenesis. Furthermore, PGC-1α regulates Sox9-dependent chondrogenesis in mesenchymal stem cells (MSCs) and chondrocytes *in vitro* (43). We therefore compared the expression levels of PGC-1α between parental and *scx*-null progenitor cells during chondrogenic differentiation. At 24 h after the induction of chondrogenic differentiation, *scx*-null progenitor cells showed marked up-regulation (∼4.0-fold increase) of PGC-1α protein compared with parental cells (Fig. 6*E*).

Finally, to investigate the direct involvement of Scx (the structural requirement for Scx) in the negative regulation of chondrogenesis, we performed rescue experiments by knockin of full-length *scx* and mutated *scx* genes into *scx*-null tenocytes. We recently established *scx*-null tenocyte lines and tenocyte lines overexpressing full-length Scx (*scx*-full) N-terminal FLAG tag fusion protein (12). In this study, we generated tenocyte lines overexpressing basic DNA-binding domain–deleted Scx (*scx*Δ*Base*) and FLAG tag fusion protein and tenocyte lines overexpressing helix-loop-helix protein interaction domain–deleted Scx (*scx*Δ*HLH*) and FLAG tag fusion protein (Fig. S10). These tenocyte clones were induced to undergo chondrogenic differentiation *in vitro* for 5 days and their expression levels of *Sox9* mRNA were compared. The absence of the *scx* gene (*scx*-null) resulted in significant up-regulation (∼1.6-fold increase; *p* < 0.05) of *Sox9* compared with *scx^flox^*^/^*^flox^* tenocytes that express Scx at physiological levels. Cells overexpressing ScxΔBase also showed significant up-regulation (∼1.4-fold increase; *p* < 0.05) of *Sox9* compared with *scx^flox^*^/^*^flox^* tenocytes. Cells overexpressing ScxΔHLH showed a slight but not significant up-regulation of *Sox9*. In sharp contrast, cells overexpressing full-length Scx showed significant down-regulation (∼2.6-fold decrease; *p* < 0.05) of *Sox9* compared with *scx^flox^*^/^*^flox^* tenocytes. Thus, taken together, these findings indicate that the promotion and maintenance of increased Scx expression levels could be essential elements to decrease the chondrogenic potential and to improve the slow-healing response to adult tendon injury.

## Discussion

In the present study, we have investigated whether Scx is a suitable molecular target for accelerating the healing response to adult tendon injury. Our comprehensive studies of adult tendon wound healing with *ScxGFP* transgenic and conditional *scx*-deficient animal models provide compelling evidence for the following propositions. 1) Scx plays indispensable roles in proper healing following adult tendon injury. 2) There is a direct link between tendon progenitor cell lineage mediated by Scx and adult tendon pathology. 3) Certain Sca-1–positive progenitor subpopulations identified in the paratenon could provide novel targets to develop strategies to facilitate tendon repair. We propose that the regulatory mechanisms underlying lineage-specific differentiation in adult tissue progenitors mediated by the transcription factor Scx shown here could translate into regenerative therapy for a broader variety of tissues or systems in the body.

Although adult tendon wound healing has been suggested to follow the same process as other organs/tissues (for reviews, see Refs. 5 and 21), as of today, the cellular and molecular mechanisms controlling adult tendon repair and remodeling following injury are still only poorly understood. Several recent lines of evidence in a full-length, central patellar tendon healing model using a lineage-tracking strategy have revealed that paratenon cells expressing α-smooth muscle actin respond to adult patellar tendon injury by turning on Scx to bridge the defect (44, 45). In the present study, we have demonstrated that paratenon cells, an Sca-1–positive and Scx-negative progenitor subpopulation, differentiate into Scx-expressing tendon cells and act as a main player in tendon repair by migrating to the wound site and actively producing ECMs to bridge the defect. Progenitor cells in the adult tendon are expected to achieve maintenance and repair following injury if properly directed toward differentiation (14). However, to date, no molecular framework exists for understanding the regulatory mechanisms by which adult tendon progenitor cells differentiate into mature tenocytes. We have identified TGF-β signaling as one of the pathways to induce differentiation of adult tendon progenitor cells into Scx-expressing tenocytes both *in vitro* and following injury.

A recent cell-based tissue engineering study using bone marrow–derived MSCs transduced with *scx* cDNA has shown that Scx plays a beneficial role in repair following adult rat tendon rupture (46). Here, we provide compelling evidence that the elimination of *scx* results in a deficiency of repair following tendon injury due to the lack of ECM assembly in paratenon-derived cells to bridge the defect. Recent *in vitro* studies have indicated that transcription of the genes pro-α2(I) collagen (*Col1a2*) (47, 48) and *Col1a1* (49) is specifically and directly controlled by Scx in tenocytes and cardiac fibroblasts. The absence of conversion from collagen type III to type I in *scx*-null wounds following tendon injury shown in this study supports the hypothesis that the transcriptional control of collagen type I is mediated by Scx. Whereas transactivation of the *Col1a2* promoter is completely abrogated by the basic Scx DNA-binding domain–deleted (*scx*Δ*Base*) mutant, the Scx helix-loop-helix protein interaction domain–deleted (*scx*Δ*HLH*) mutant shows a lesser phenotype, suggesting that the Scx helix-loop-helix protein interaction is not absolutely required for full Scx activity (47, 48). These observations support our findings showing the relationship of full-length/mutated *scx* genes to *Sox9* mRNA regulation during chondrogenesis.

The transcription factor Sox9 and TGF-β–mediated signaling are necessary for chondrogenic differentiation in MSCs *in vitro* (38, 50). PGC-1α has recently been shown to act as a coactivator for Sox9 that directly interacts with Sox9 and promotes Sox9-dependent transcription activity during chondrogenesis. Although Sox9 is expressed not only in chondrogenic but also in nonchondrogenic tissues, PGC-1α cooperates with Sox9, and they have a synergistic effect on the up-regulation of chondrogenesis-specific genes such as collagen type II α1 chain followed by TGF-β–induced chondrogenesis in MSCs *in vitro* and limb development during embryogenesis (43). This evidence is consistent with our observations demonstrating the significantly enhanced chondrogenic potential of *scx*-null tendon progenitor cells with up-regulated Sox9 and PGC-1α expression *in vitro* and the formation of cartilage-like tissue in *scx*-null wounds *in vivo* following tendon injury. Our findings suggest that there are functional links between Scx and PGC-1α expression and the involvement of Scx in negative regulation in TGF-β/Sox9–mediated chondrogenesis.

The wound in *scx*-null Achilles tendon following partial transection injury results in the spontaneous formation of chondroid foci and ectopic ossification. An earlier study shows that, upon forced overexpression of Sox9 in tenocytes, ectopic cartilage formation is preferentially observed in dense connective tissues, including tendons/ligaments (51). During tooth development, overexpression of the *scx* gene in periodontal ligament cells results in significant inhibition of mineralization by osteoinduction, and knockdown of the *scx* gene results in marked up-regulation of the osteogenic transcription factor osterix, suggesting that Scx counteracts osterix-driven osteogenesis (52). These findings document the counteracting effects of lineage-specific transcription factors on cellular differentiation and support the hypothesis that Scx is the key transcription factor in the prevention of chondrogenesis and osteogenesis. Interestingly, a very recent study provides evidence that the absence of the tendon-specific transcription factor Mohawk causes spontaneous heterotopic ossification of Achilles tendon in rats (53).

From the current findings, we propose the following scenario. Following tendon injury, the reduction of physical force from skeletal muscle results in the down-regulation of Scx or the failure of its induction in tendon progenitors. This could cause significantly decreased production of ECM to bridge the defect, as a result of which these progenitors could possess high condrogenic potential, and consequently generate chondroid foci and ectopic ossification. This phenomenon has biological relevance to the slow-healing response to tendon injury. The promotion of increased Scx expression levels and the maintenance of Scx-positive cells could be promising strategies to improve the slow-healing response to adult tendon injury. It would be of interest to evaluate tendon mechanoproperties following injury to further address the functional role of Scx. The incidence of tendon injury has increased in recent years as a result of the aging of the population (5, 21, 54). Heterotopic tendon mineralization (ossification or calcification), a frequent complication following trauma or surgery, is a significant medical problem because it is associated with pain and dysfunction (55). Despite all efforts, current treatment modalities in adult tendon injury are still not optimal, and alternative strategies are needed (56). Because there is a critical need for repair strategies in adult tendon injuries that provide adequate enhancement of patients' healing potential, the present study could provide a foundational paradigm for evaluating a number of modified or alternative therapeutic approaches to enhance the tendon environment for healing using tendon progenitors.

## Materials and methods

### Mice

To visualize tenocytes *in vivo*, *ScxGFP* transgenic mice were used. The *ScxGFP* transgene features tendon-specific regulatory sequences from the mouse *scx* gene that drive the expression of GFP. Like the endogenous *scx* gene, this transgene specifically marks tenogenic cells and tenocytes (9). To generate mice with a tissue-specific deletion of the *scx* gene, *scx^flox^*^/^*^flox^* mice were used (10). *scx^flox^*^/^*^flox^* mice displayed no obvious abnormalities, were fertile, and had a normal lifespan. Tomato red transgenic mice and *ROSA26* (41) mice were from The Jackson Laboratory.

All animal experiments were approved by the Institutional Animal Care and Use Committee and by the local ethical review committee and licensed by the UK Home Office (70/9039: T. Sakai). All mice were maintained and bred at the animal facility in accordance with institutional guidelines. Mice were regularly monitored and had free access to standard mouse chow and water. At least five mice were used per group in each time point and the evaluated phenotypes.

### Antibodies and reagents

The following antibodies were used: rabbit polyclonal antibodies against mouse Scx (12), collagen type I (Chemicon), collagen type II (Chemicon), bovine N-propeptide of collagen type III (23), and aggrecan (Chemicon); goat polyclonal antibody against fibrinogen (Nordic Immunological Laboratories); rat monoclonal antibodies against Sca-1 (BD Pharmingen), PECAM-1 (BD Pharmingen), CD18 (BD Pharmingen), and mouse integrin β1 (clone 9EG7), which recognizes the ligand-inducible activated integrin β1 (57) (BD Pharmingen); rabbit polyclonal antibody against phospho-Smad2C, which specifically recognizes the phosphorylated C-terminal serine 465/467 of Smad2 (a kind gift from Dr. Koichi Matsuzaki, Kansai Medical University, Japan); rabbit polyclonal antibodies against tyrosine 397–phosphorylated focal adhesion kinase (BioSource), PGC-1α (Santa Cruz Biotechnology); rabbit mAb against Ki67 (Lab Vison); mouse monoclonal antibodies against FLAG M2 (Sigma), β-actin (clone AC15; Sigma), and heat shock protein 70 (HSP70; Santa Cruz Biotechnology); and Cy3-conjugated donkey anti-rabbit and anti-rat IgG and peroxidase-conjugated donkey anti-mouse and anti-rabbit IgG (Jackson ImmunoResearch Laboratories). Alexa Fluor 568 donkey anti-goat IgG was from Invitrogen. Rabbit polyclonal antibodies against fibromodulin and COMP were kind gifts from Dr. Dick Heinegård (Lund University, Sweden). The following cytokines/growth factors were used: recombinant human TGF-β1, -β2 and -β3; recombinant mouse GDF5, -7, and -8; recombinant human BMP2; recombinant mouse VEGF; recombinant human FGF4; recombinant mouse IGF-1; and porcine PDGF (all from R&D Systems). An adenovirus encoding *Cre* recombinase was a kind gift from Dr. Dusko Illic (University of California at San Francisco).

### Adult mouse Achilles tendon partial transection model

We developed a simple and reproducible partial transection model to minimize postoperative complications. Mice (10–12 weeks old) were anesthetized, and the Achilles tendon was exposed through an anterolateral skin incision (∼1 cm long) slightly above the calcaneus. Then a partial transection (0.3 mm in width) at ∼2 mm proximal from the calcaneal insertion was created in the right Achilles tendon without massive bleeding using a number 11 surgical blade (Feather Safety Razor Co.; Fig. 1*B*). As an internal control, sham operations were performed on the left Achilles tendon. Then the skin was closed using 7-0 Ethilon sutures (Ethicon). All surgical procedures were performed by a single surgeon to enhance consistency. Mice were allowed to resume normal cage activity until sacrifice.

### Local injection of adenovirus-Cre into wounded site of adult Achilles tendon

To inactivate the *scx* gene in an injured tendon, we used an adenovirus encoding *Cre* recombinase as described previously (12). To determine the most appropriate amount of virus for *Cre* transduction and successful excision of the *scx^flox^*^/^*^flox^* gene in our hands *in vivo*, initial studies were performed using mice harboring the well characterized *ROSA26^flox^*^/^*^flox^-LacZ* gene. This gene expresses a bifunctional LacZ/neomycin phosphotransferase protein in any cell (and cell progeny) once recombined by *Cre* (41). We performed single injections of adenovirus-*CreGFP* (1 × 10^8^, 5 × 10^8^, and 1 × 10^9^ m.o.i. in 10 μl of PBS) into the right Achilles tendon of 10–12-week-old *ROSA26^flox^*^/^*^flox^-LacZ* and WT mice. As an internal control, PBS alone (10 μl) was injected into the left Achilles tendon. We confirmed efficient *Cre*-mediated gene recombination by LacZ staining in tenocytes at 1 week after injection using a dose of 1 × 10^9^ m.o.i. of adenovirus-*Cre* per mouse (Fig. S9A). No endogenous LacZ expression was found in WT mice (data not shown).

We also gave a single injection of 1 × 10^9^ m.o.i. adenovirus-*Cre* into the Achilles tendon of 10–12-week-old *scx^flox^*^/^*^flox^* mice. Considerably decreased expression of Scx protein in tenocytes was observed by immunohistochemistry at 7 days after injection (Fig. S9B), indicating that adenovirus-*Cre* at of 1 × 10^9^ m.o.i. results in the efficient deletion of floxed alleles in the *scx* gene. No decrease in tenocyte numbers was observed in adenovirus-GFP–injected control tendons (data not shown).

### Bone marrow transplantation analysis

To study the contribution of bone marrow–derived cells to tendon wound healing, bone marrow transplantation was performed to introduce Tomato red–labeled bone marrow cells into WT mice. Adult C57B6 mice (10–12 weeks of age) were irradiated at a controlled, sublethal dose of 10 grays (1000 rads) using a γ irradiator (^137^Cs Shepherd irradiator). The mice were then anesthetized, and 2 × 10^6^ bone marrow cells isolated from tomato red transgenic mice were injected into recipient mice from the retroorbital venous sinus. The recipient mice were kept for a further 6 weeks and then used for the analysis of Achilles tendon injury.

### Isolation, generation, and characterization of mouse adult parental and scx-null tendon progenitor cell lines

We generated parental and *scx*-null progenitor cell lines from adult mouse Achilles tendon. Briefly, primary tenocytes from adult *scx^flox^*^/^*^flox^*/*ScxGFP* mouse Achilles tendon under a *p53*- and *p21*-null genetic background were isolated as described previously (11). Because CD90.2 is known to be expressed in tendon stem/progenitor cells but not in bone marrow stromal cells (14), we next performed fluorescence-activated cell sorting (FACS) using anti-CD90.2 and subsequent Sca-1 antibodies. Then positive cells were cloned, and several immortalized clones were generated. Several of the clones were treated with a *Cre*-transducing adenovirus to delete the floxed *scx* genes (12), and the deletion of *scx* alleles was confirmed by PCR (Fig. S7A). Both parental *scx^flox^*^/^*^flox^* and *scx*-null progenitor lines showed similar fibroblastic morphology (Fig. S7B).

### Self-renewal and cell differentiation analyses and effects of cytokines/growth factors on ScxGFP induction in progenitor cells

To explore the clonogenic potential of established progenitor cell lines, a cfu assay was performed. Briefly, cells were seeded in 10-cm Petri dishes at a density of 10 cells/cm^2^. After 10 days, colonies formed were visualized by staining with 0.5% crystal violet. cfu efficiency was calculated with the following formula: cfu (%) = (Number of colonies formed/Number of cells plated) × 100.

Parental and *scx*-null progenitor cells were differentiated into three different mesodermal lineages as described previously (58) with some modifications. To introduce adipogenic differentiation, cells were cultured for 21 days in DMEM high glucose supplemented with 10% FBS, 1 μm dexamethasone, 0.2 mm indomethacin, 1 mm 3-isobutyl-1-methylxanthine, and 0.1 mg/ml insulin (all reagents were from Sigma). Then lipid vacuoles were visualized by Oil Red O staining using a standard protocol. To introduce chondrogenic differentiation, cells (3 × 10^5^) were seeded in M-shaped 96-well plates (PrimeSurface 96M, Sumitomo Bakelite Co., Japan) to form cell aggregates (pellet culture), and cells were further cultured for up to 21 days in differentiation medium (composed of DMEM high glucose supplemented with 10 μm dexamethasone, 1 nm sodium pyruvate (Invitrogen), 50 μg/ml l-ascorbic acid 2-phosphate (Sigma), 1% insulin, transferrin, and sodium selenite mixture (Invitrogen), and 10 ng/ml TGF-β1 (R&D Systems)). Then cell pellets were fixed in 4% paraformaldehyde in PBS (pH 7.2) and embedded in OCT compound (Tissue-Tek, Sakura Finetek), and then cryosections were prepared and stained with toluidine blue and Safranin O. In some experiments, protein and total RNA were isolated and used for Western and real-time PCR analysis, respectively. To induce osteogenic differentiation, cells (4 × 10^3^ cells/cm^2^) were seeded in 12-well plates and cultured for 10 days in osteogenic medium (DMEM high glucose supplemented with 10% FBS, 10 mm β-glycerophosphate, 100 nm dexamethasone, 50 μg/ml l-ascorbic acid 2-phosphate, and 200 ng/ml BMP2 (R&D Systems)). The extent of osteogenic differentiation was determined by Alizarin Red staining.

For quantification of osteogenic areas, images were captured with the same gain, offset, magnitude, and exposure time. A minimum of four different images were randomly selected, and their intensities were quantified using ImageJ software (version 1.48; National Institutes of Health) (59). To quantify cellular ScxGFP intensity in response to cytokines/growth factors in live progenitors, the mean intensity in each cell was measured, and the average GFP intensity per cell (fluorescent units) was calculated as described previously (11).

### Generation of scleraxis-null and scleraxis-expressing tenocyte lines

Isolation of tenocytes from the Achilles tendon of adult mice on a *scx^flox^*^/^*^flox^*/*ScxGFP*/*p53*- and *scx^flox^*^/^*^flox^*/*ScxGFP*/*p21*-null genetic background was performed as described (11). Then *scx*-null and its parental cell lines and full-length Scx and N-terminal FLAG tag fusion protein–overexpressing tendon lines were established as described previously (12). To generate murine tenocyte lines expressing mutated Scx (Scx with the basic DNA-binding domain deleted (ScxΔBase) and Scx with the helix-loop-helix protein interaction domain deleted (ScxΔHLH)), we generated an Scx and FLAG tag fusion protein expression construct. FLAG tag was inserted at the N-terminal end (upstream of the start codon) or the C-terminal end (upstream of the stop codon) of cDNA encoding *scx* and cloned in lentiviral pCDH-CMV-MCS1-Puro expression vector (pCDH-CMV-MCS-Puro_ScxFLAG, pCDH-CMV-MCS-Puro_ScxΔBase, and pCDH-CMV-MCS-Puro_ScxΔHLH; System Biosciences). For lentivirus production, HEK293T cells were transfected with pCMV-VSV-G (Addgene plasmid 8454), pCMV-dR8.2 dvpr (Addgene plasmid 8455), and pCDH-CMV-MCSPuro_full-length ScxFLAG/mutated ScxFLAG or pCDH-CMV-MCS-Puro (mock) using Lipofectamine (Invitrogen) (60). Then lentiviral infection was performed in *scx*-null tendon cell lines. To generate stable clones, selection with 10 μg/ml puromycin was started at 72 h after transfection, and the surviving clones were isolated and expanded.

### Histological analysis, immunohistochemistry, and immunofluorescence

To visualize the ScxGFP signal, Achilles tendons were fixed in 4% paraformaldehyde in PBS, cryoprotected in PBS containing 15% sucrose, and frozen in OCT compound. Cryosections (10 μm thick) were prepared, mounted in Vectashield mounting medium containing DAPI (Vector Laboratories) and analyzed by fluorescence microscopy. After fluorescence analysis, each section was stained with hematoxylin and eosin (H&E) and observed under bright-field illumination as described previously (11). Histological analysis, LacZ staining, and immunohistochemistry and immunofluorescence studies were performed as described previously (61, 62).

### Real-time PCR

Real-time PCR was performed as described elsewhere (11, 63). The following primers were used: *scx* forward, 5′-GAGACGGCGGCGAGAAC-3′; *scx* reverse, 5′-TTGCTCAACTTTCTCTGGTTGCT-3′; *sox9* forward, 5′-CGGCTCCAGCAAGAACAAG-3′; *sox9* reverse, 5′-TGCGCCCACACCATGA-3′; collagen type II forward, 5′-AGAACAGCATCGCCTACCTG-3′; collagen type II reverse, 5′-CTTGCCCCACTTACCAGTGT-3′; *runx2* forward, 5′-GCCACTTACCACAGAGCTATT-3′; *runx2* reverse, 5′-GAGGCGATCAGAGAACAAACT-3′; fibromodulin forward, 5′-AGCAGTCCACCTACTACGACC-3′; fibromodulin reverse, 5′-CAGTCGCATTCTTGGGGACA-3′; 18S rRNA forward, 5′-GGCGACGACCCATTCG-3′; and 18S rRNA reverse, 5′-ACCCGTGGTCACCATGGTA-3′. All samples were analyzed at least in triplicate. After the reactions, the specificity of amplification in each sample was confirmed by dissociation analysis, showing that each sample gave a single melting peak. Relative mRNA levels were normalized to the level of 18S rRNA.

### Western blot analysis

Western blot analyses were performed as described elsewhere (23, 62). In some immunoblot analyses, samples were transferred onto an Immobilon-FL polyvinylidene fluoride (PVDF) membrane (Millipore Corp.) and probed with primary and IRDye 800CW- or IRDye 680-conjugated secondary antibodies (LI-COR Biosciences). Immunoreactive bands were detected using the Odyssey IR Imaging System (LI-COR Biosciences).

### Micro-CT analysis

The eXplore Locus micro-CT (GE Healthcare), a gantry-based scanner with a rotating X-ray source and detector (fixed anode with tungsten target source, operating from 40 to 80 kV peak at 0.5-mA maximum current), was used for the analysis. Live mice were placed on a linearly encoded, computer-controlled heated bed that translated (250-mm travel) into the gantry. With a rotational precision of 0.05°, up to 1000 projections were collected in step-and-shoot mode using a 16-bit charge-coupled device camera (1000 × 1000) and one of three resolution modes: 26 μm (40-mm-diameter field of view), 45 μm (88-mm-diameter field of view), and 93 μm (88-mm-diameter field of view). During acquisition, continuous-flow isoflurane anesthesia was administered via a nose-cone adapter (Summit Anesthesia), and the physiological state of the animal (heart rate, temperature, and respiration) was monitored via electrodes and strain gauges (Spin Systems). For situations in which the scanning area was adversely affected by breathing-induced motion artifacts, acquisition was directly gated using respiratory signals. Following acquisition, reconstruction of projection data was performed on a PC Unix cluster using a multithreaded reconstruction algorithm.

### Data presentation and statistical analysis

All experiments were performed at least in triplicate on separate occasions, and the data shown were chosen as representative of results consistently observed. Results are presented as means ± S.D. Differences between groups were analyzed using the two-sided Student's *t* test on raw data. In cases where more than two groups were compared, Dunnett's post hoc test was used. A *p* value of <0.05 was considered significant.

## Author contributions

T. Sakabe and T. Sakai conceptualization; T. Sakabe, K. S., T. M., A. S., and T. Sakai formal analysis; T. Sakai supervision and funding acquisition; T. Sakabe, K. S., T. M., A. S., N. F., and R. S. investigation; T. Sakai writing-original draft; T. Sakabe, K. S., T. M., and T. Sakai project administration; T. Sakai writing-review and editing; A. S., N. F., R. S., T. Sasaki, and T. Sakai resources.

## Supplementary Material

Supporting Information

## Footnotes

2 The abbreviations used are:

ECM: extracellular matrix
MSC: mesenchymal stem cell
PGC-1α: peroxisome proliferator-activated receptor γ coactivator-1α
Scx: Scleraxis
Sca-1: stem cell antigen-1
Sox9: SRY-box 9
PPAR: peroxisome proliferator–activated receptor
PECAM: platelet endothelial cell adhesion molecule
COMP: cartilage oligomeric matrix protein
BMSC: bone marrow–derived stromal cell
pSmad: phospho-Smad
*runx2*: runt-related gene-2
m.o.i.: multiplicity of infection
CT: computed tomography
HLH: helix-loop-helix.
