# Supplementary material for: Transcription factor scleraxis vitally contributes to progenitor lineage direction in wound healing of adult tendon in mice
Source: J Biol Chem. 2018 Mar 5;293(16):5766–80. doi: 10.1074/jbc.RA118.001987 (PMC5912447; doi:10.1074/jbc.RA118.001987)
Supplement: Supporting Information [file supp_293_16_5766__index.html]

Transcription factor scleraxis vitally contributes to progenitor lineage direction in wound healing of adult tendon in mice — Scleraxis in adult tendon wound healing — Transcription factor scleraxis vitally contributes to progenitor lineage direction in wound healing of adult tendon in mice — Scleraxis in adult tendon wound healing — Supporting Information 

# Transcription factor scleraxis vitally contributes to progenitor lineage direction in wound healing of adult tendon in mice

## Supporting Information

- Supporting information - Supporting information
