# Supplementary material for: Transcription factor scleraxis vitally contributes to progenitor lineage direction in wound healing of adult tendon in mice
Source: J Biol Chem. 2018 Mar 5;293(16):5766–80. doi: 10.1074/jbc.RA118.001987 (PMC5912447; doi:10.1074/jbc.RA118.001987)
Supplement: Supporting Information [file supp_RA118.001987_135420_1_supp_84693_p4t95b.docx]

**Supporting Information**

**Transcription factor scleraxis vitally contributes to progenitor lineage direction in wound healing of adult tendon in mice**

**Tomoya Sakabe, Keiko Sakai, Toru Maeda, Ataru Sunaga, Nao Furuta, Ronen Schweitzer, Takako Sasaki, and Takao Sakai**

**(Supplementary Figure 1 – Figure 10)**


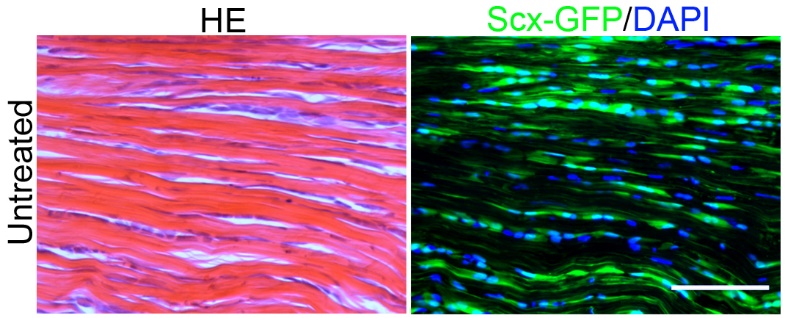


**Supplementary Fig. 1.** Normal morphology, and robust ScxGFP expression in Achilles tendon of adult 10-week-old *ScxGFP* mice. Scale bar = 100 μm.


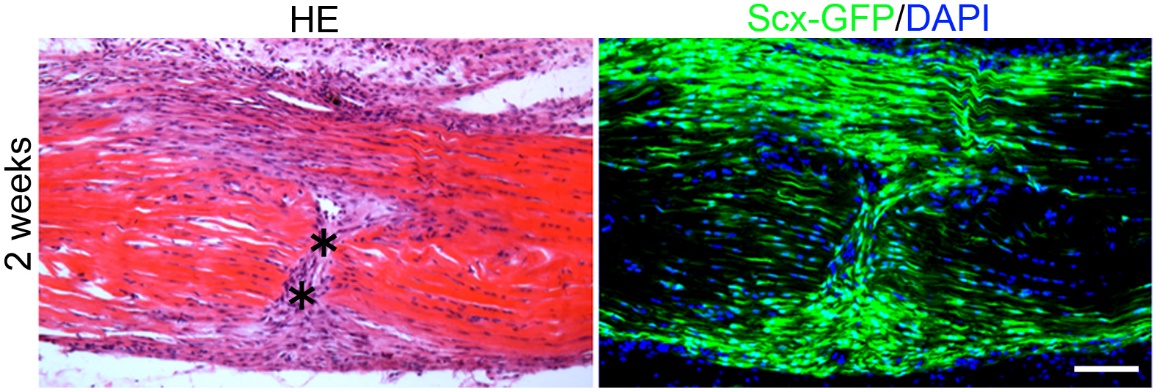


**Supplementary Fig. 2.** Histological analysis of Achilles tendon in the partial transection model at 2 weeks after tendon injury (at low magnification). HE staining (left panel) and the same area with GFP/UV filter (right panel; green, ScxGFP; blue, nuclear staining with DAPI). Asterisks in HE sections indicate wounds. Scale bar = 100 μm.


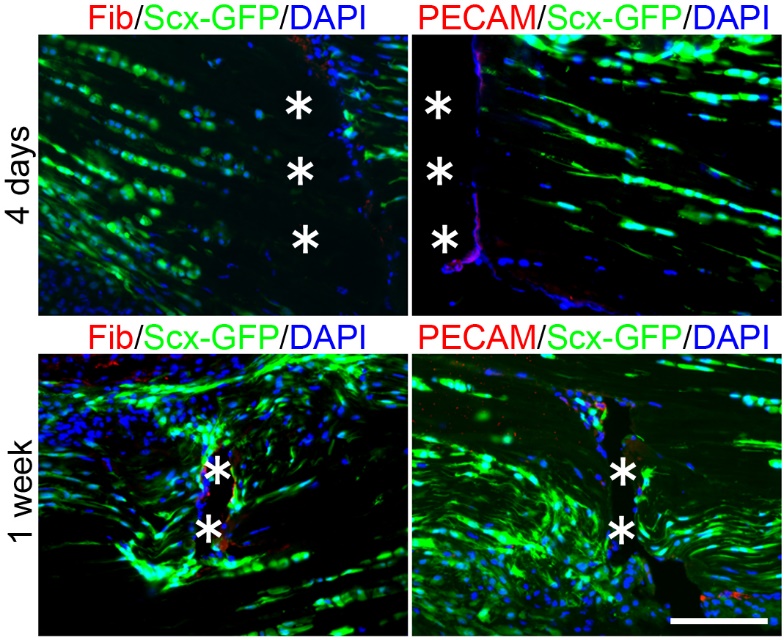


**Supplementary Fig. 3.** Tissue distribution of fibrinogen and PECAM at 4 days and 1 week after tendon injury. Triple immunofluorescence staining for fibrinogen (Fib) or PECAM (red)/ScxGFP (green)/ DAPI (blue). Asterisks indicate wounds. Scale bar = 100 μm.


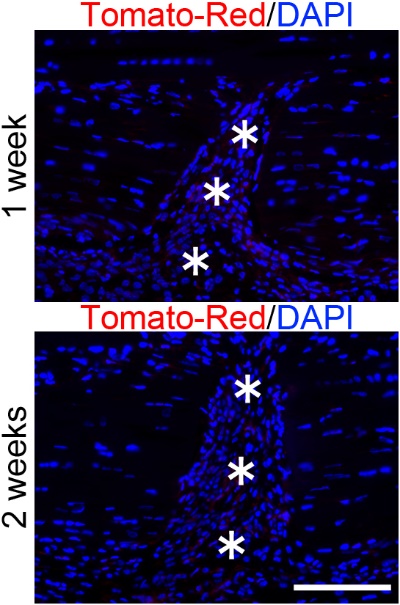


**Supplementary Fig. 4.** Tissue distribution of bone-marrow-derived cells at 1 and 2 weeks after tendon injury in mice treated with bone marrow transplantation. Double immunofluorescence staining for bone-marrow-derived cells (Tomato-Red)/DAPI (blue). Asterisks indicate wounds. Scale bar = 100 μm.


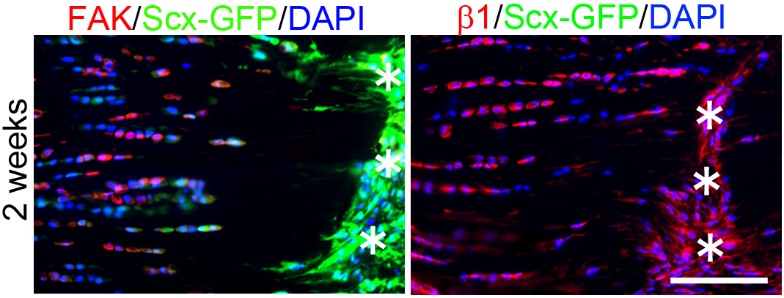


**Supplementary Fig. 5.** Tissue distribution of focal adhesion kinase (FAK) and β1 integrin positive cells at 2 weeks after injury. Triple immunofluorescence staining for tyrosine 397-phosphorylated FAK (pFAK-Tyr397) or ligand inducible active β1 integrin (red)/ScxGFP (green)/ DAPI (blue). Asterisks indicate wounds. Note that sections for activated β1 integrin staining were fixed in methanol, resulting in the loss of ScxGFP signals. Scale bar = 100 μm.


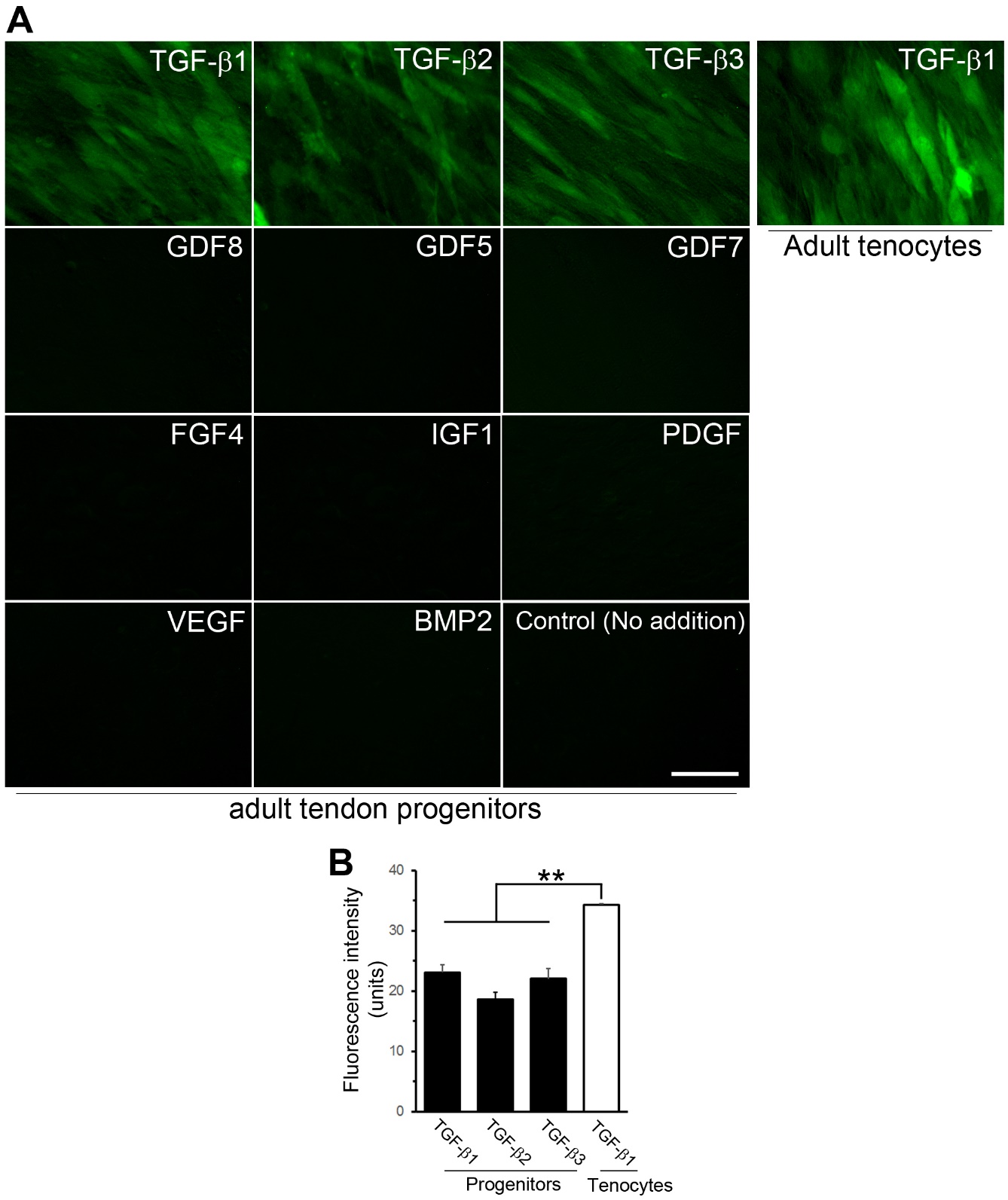


**Supplementary Fig. 6.** Effects of cytokines and growth factors on ScxGFP expression in adult progenitor cell lines *in vitro*.

**(A)** Tendon progenitor cells were cultured for 48 hr, then cytokines/growth factors (TGF-β1, -β2 and -β3, 2 ng/mL; GDF8, 500 ng/mL; GDF5, 2.0 μg/mL; GDF7, 1.0 μg/mL; FGF4, 20 ng/mL; IGF-1, 50 ng/mL; PDGF, 20 ng/mL; VEGF, 100 ng/mL; and BMP2, 200 ng/mL) were added for a further 7 days. The addition of TGF-β1, -β2 and -β3 resulted in the induction of ScxGFP expression. None of the other cytokines/growth factors had any effect on the regulation of ScxGFP expression. Control (no addition), DMEM containing 1% FBS. Scale bar = 100 μm.

**(B)** Analysis of ScxGFP intensity in progenitors and tenocytes treated with TGF-βs. The intensity is shown relative to the control value of 100 (treated with TGF-β1). Error bars represent the standard deviation (*n* = 6 for each group). The intensity of ScxGFP in progenitors is similar after treatment with any of the three TGF-βs. The intensity of ScxGFP in tenocytes treated with TGF-β1 is significantly higher than that in progenitors. **, *P* < 0.01.


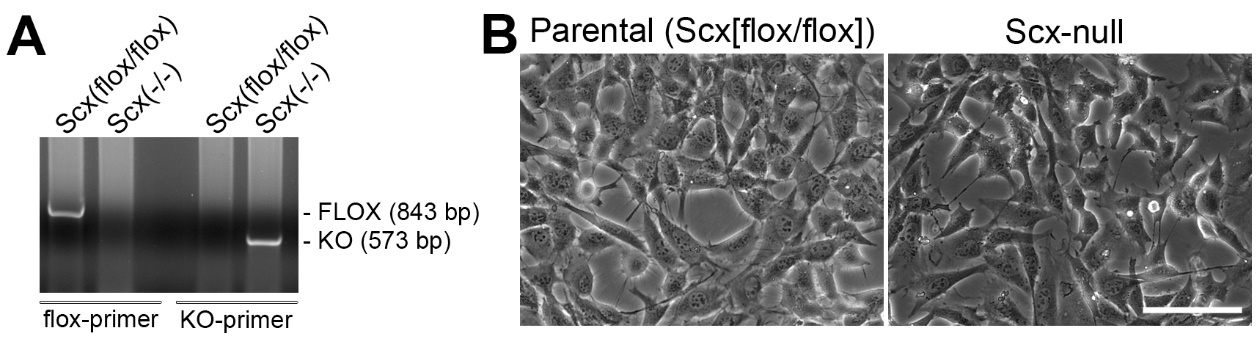


**Supplementary Fig. 7.** Generation of *scx-*null tendon progenitor cell lines from adult Achilles tendon.

**(A)** PCR analysis of adult tendon progenitor clones before and after treatment with a *Cre*-transducing adenovirus to delete the *scx*-floxed genes. PCR products for *scx*-floxed (843 bp) and *scx*-null (573 bp) are indicated.

**(B)** Cellular morphology of *scx-*null and its parental [*scx(flox/flox)*] progenitor cells under phase-contrast microscopy. Scale bar = 100 µm.





**Supplementary Fig. 8.** *scx*-null progenitor cells show a similar osteogenic activity to parental cells in response to BMP2.

**(A)** Western blot analysis of phospho-Smad1/5/8 (pSmad1/5/8) protein expression in *scx*-null and parental (control) progenitor cells at 2 and 24 hr after treatment with 200 ng/mL BMP-2 (osteogenesis medium). The band intensities were measured by densitometry and the intensity of the control sample band at 2 hr after BMP-2 treatment was set to 1. Each value is shown relative to this control value. The positions of molecular weight marker are indicated.

**(B)** Real-time PCR analysis of *runx2* mRNA level at 72 hr after treatment with 200 ng/ml BMP-2 (osteogenesis medium). Relative mRNA expression level is shown relative to the control value of 1. Error bars represent the standard deviation (*n* = 3 for each group). n.s., not significant.


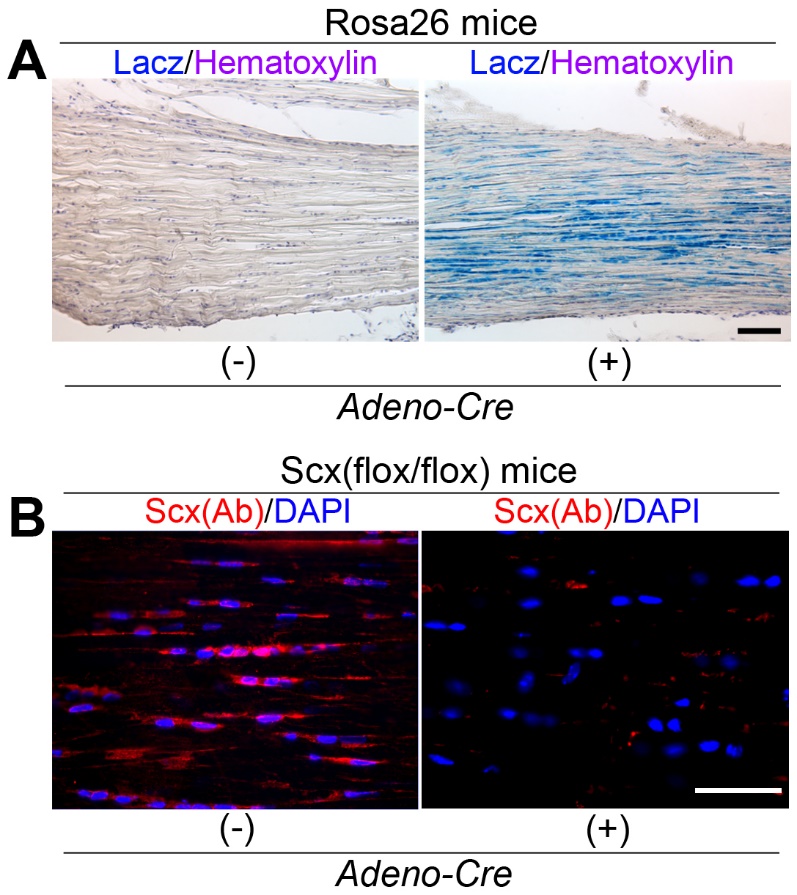


**Supplementary Fig. 9.** Inactivation of *Scx* from adult tenocytes by local injection of adenovirus-*Cre*.

**(A)** LacZ staining of the Achilles tendon in Rosa26 mice at 1 week after local injection of adenovirus-*Cre* or PBS into Achilles tendon. Double staining for LacZ and hematoxylin. Scale Bar = 100 μm.

**(B)** Immunostaining for Scx (Scx[Ab]) in the Achilles tendon of *scx(flox/flox)* mice at 1 week after local injection of adenovirus-*Cre* or PBS. The expression of *Scx* is markedly decreased in adenovirus-*Cre*-treated Achilles tendon. Scale bar = 100 μm.


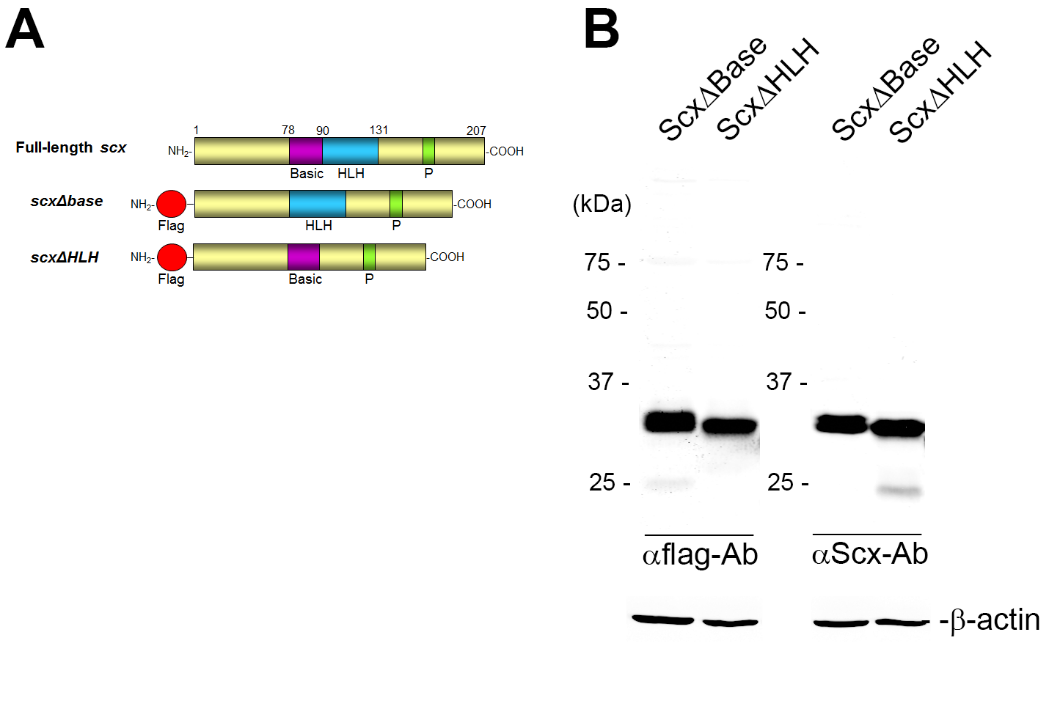


**Supplementary Fig. 10.** Generation of tenocyte lines overexpressing mutated Scx.

**(A)** Diagram of full-length *scx* and mutated-*scx* molecules. Basic, basic DNA-binding domain; Flag, Flag-tag; HLH, helix-loop-helix protein interaction domain; P, proline rich domain.

**(B)** Western blot analysis of lysates from flag-tag/mutated Scx fusion protein transfected cells (ScxΔBasic, overexpressing the basic DNA-binding domain-deleted Scx and flag-tag fusion protein; ScxΔHLH, overexpressing the helix-loop-helix protein interaction domain-deleted Scx and flag-tag fusion protein). After protein transfer, the membrane was cut and performed immunoblot using anti-flag-tag and anti-Scx antibodies. The position of a molecular mass marker (kDa) is indicated. β-actin served as a loading control. The molecular size of the mutated ScxΔBasic and ScxΔHLH detected with anti-Scx antibody is completely identical to that detected with anti-flag antibody.
